# Supplementary material for: Impact of non-agricultural employment on industrial structural upgrading -Based on the household consumption perspective
Source: PLoS One. 2024 Feb 21;19(2):e0294333. doi: 10.1371/journal.pone.0294333 (PMC10881005; doi:10.1371/journal.pone.0294333)
Supplement: Attachment — Submitted filename: Response to Reviewers.docx [file pone.0294333.s002.docx]

Dear Editors and Reviewers:

Thank you very much for the opportunity to revise our manuscript. We appreciate the comments and noting of the significance of this work from the Reviewers and the Editor. Those comments are valuable and helpful for revising and improving our paper, as well as the important guiding significance to our researches. We have revised our manuscript according to these comments and please find our point-by-point replies to Editors and Reviewers comments. The main corrections in the paper and the responds to the reviewer’s comments are as following:

Responds to the reviewer’s comments:
Reviewer #1:

1.Response to comment:“**It is necessary to be included further references .**”

Response: We sincerely appreciate the valuable comments. We have checked the literature carefully and added more references into the INTRODUCTION part in the revised manuscript.

2.We didn’t see comment with serial number 2 in the email we received.

3. Response to comment:“**The discussion should be improved. There needs to be more comparative analysis with other studies.**”

Response: We think this is an excellent suggestion. We have analyzed and compared the existing research on human capital and industrial upgrading in lines 32-34 on the third page. However, there is less research in this aspect at present, and it will be supplemented in time in subsequent research.

4.Response to comment: “**Conclusions should be improved in a more concisely way and compared with similar studies.**”

Response: Thanks for your suggestion. We have revised the research conclusion to a more concise expression.

Reviewer #2:

1.Response to comment:“ **One noticeable concern is the prolonged sentences scattered throughout the paper. These can make comprehension somewhat daunting.** ”

Response: We tried our best to improve the manuscript and made some changes to the manuscript. Here we did not list the changes but marked in red in the revised paper. We appreciate for Reviewer’s warm work earnestly and hope that the correction will meet with approval.

2.Response to comment:” **The manuscript could benefit from a section detailing the research's limitations, any uncertainty in the results, and the future work designed to address these gaps.**”

Response: Thanks for your suggestion. We have added the limitations of existing research and prospects for future research.

3&4.Response to comment “**Moreover, in the current global context, the omission of any mention of climate change—a pressing concern—is glaring.**” and “**Given the profound repercussions of the COVID-19 pandemic, its absence from the discussion is notable.**”

Response: As suggested by the reviewer, we have added more references to support our research. We have checked the literature carefully and added more references on climate change and COVID-19 into the INTRODUCTION part in the revised manuscript.

All revisions have been made with respect to the feedback received, and I hope they meet your expectation. Once again, thank you for your constructive critique. In conclusion, I hope the revised manuscript will be deemed suitable for publication. I eagerly await your further comments or decision.

Sincerely,

Jian Cao

Email:1065096790@qq.com
